# Advances in the study of depression and anxiety in Parkinson’s disease: A review

**DOI:** 10.1097/MD.0000000000041674

**Published:** 2025-03-07

**Authors:** Zhonglong Wang, Hongkai Wei, Yanhong Xin, Wei Qin

**Affiliations:** a Shandong Daizhuang Hospital, Jining, China; b Department of Psychiatry, Shandong Mental Health Center Affiliated to Shandong University, Shandong, Jinan, China.

**Keywords:** anxiety, depression, Parkinson’s disease, research advances

## Abstract

Motor symptoms are central to diagnosing Parkinson’s disease (PD), but depression and anxiety significantly impact the prognosis and course of PD. For many PD patients, these mental health issues may be the most crucial determinants of quality of life. This study uses an interdisciplinary approach to provide an in-depth understanding of the pathogenesis, diagnostic methods, and therapeutic strategies for depression and anxiety in PD, incorporating neuroscience, psychiatry, and psychology. It aims to offer theoretical support for developing personalized medicine and precise treatments, as well as insights into future research directions. The objective of this study was to systematically sort out the research progress of PD with depression and anxiety and to provide a basis for clinical optimization of treatment strategies. Extensive searches of relevant domestic and international databases, including PubMed, Web of Science, Embase, Cochrane Library, CNKI, etc., were conducted to screen the high-quality research literature on the treatment of PD depression and anxiety in the last decade and to conduct comprehensive analyses and generalizations. Pharmacological treatments, including 5-hydroxytryptamine reuptake inhibitors, norepinephrine system agents, and dopamine agonists, showed some efficacy but with individual differences and side effects. Psychotherapies such as cognitive-behavioral therapy and group counseling improved patient mood. Neuromodulation techniques like deep brain stimulation also showed promise in refractory cases. The combined application of multiple therapeutic approaches shows good prospects in the treatment of depression and anxiety in PD, but in-depth studies are still needed to determine the optimal treatment plan while focusing on individual patient differences to achieve precise treatment.

## 1. Introduction

Parkinson’s disease (PD) is the second most common neurodegenerative disease, and its incidence is increasing every year.^[1]^ In recent years, with the increasing understanding of PD, the impact of depression and anxiety on quality of life is more significant and severe compared with other motor and nonmotor symptoms in PD patients.^[2]^ However, there are still many controversies and unresolved questions about the pathogenesis, diagnostic criteria, and effective treatment strategies for PD depression and anxiety. In-depth research on PD depression and anxiety not only helps to improve the quality of life of patients and reduce the burden on families and society but also provides an important theoretical basis and practical experience for the development of the field of neuropsychiatry. Therefore, it is of great clinical and scientific significance to systematically sort out and summarize the research progress of PD depression and anxiety.

To better understand PD depression and anxiety, we searched Chinese and English databases: PubMed, Web of Science, Embase, Cochrane Library, CNKI, etc. The Chinese search terms were “anxiety,” “depression,” “psychiatric,” “PD,” “PD autonomic,” “non-motor symptoms,” “PD autonomic,” “non-motor symptoms” and “PD anxiety.” “psychiatric,” “PD,” “PD autonomic,” “non-motor symptoms,” “recent advances,” “PD depression diagnosis,” “PD anxiety diagnosis,” “PD depression therapy,” “PD anxiety treatment,” “anxiety,” “depressed,” “spirit mental disease,” “Parkinson′s disease,” “autonomic nerve ‘,’ non-motor symptoms,” “Latest progress,” “Diagnosis of Parkinson’s Depression,” “Diagnosis of Parkinson’s Anxiety,” “Treatment of Parkinson’s Depression,” “Treatment of Parkinson’s Anxiety.” References to relevant articles were also searched manually. The search timeframe was from the construction of the database to July 2024. After a systematic review and analysis to narrow down the search to include literature, studies were mainly published in the last 5 years, with a maximum of 30 years. A brief review of PD depression and anxiety is presented in the hope that it will provide a reference for future exploration and development of depression and anxiety interventions for PD patients and further improve the quality of life and physical and mental health of PD patients.

## 2. Epidemiological characteristics of depression and anxiety in PD

Depression is an independent and highly prevalent non-motor symptom in PD that appears in the early stages and persists throughout the disease. In addition, a number of clinical features as well as motor and nonmotor symptoms appear to be associated with depression and negatively impact quality of life. Through a meta-analysis encompassing 129 trials with a total of 38,304 participants, it was found that the prevalence of depression in PD patients was 38%. Major depression was found in about 17% of PD patients.^[3]^ Up to 70% of outpatients with mid- to late-stage PD have no dementia and are seen to be depressed with other neuropsychiatric symptoms.^[4]^The characteristics of depression in PD are as follows: depression and anxiety disorders frequently coexist and can precede motor symptoms in PD.^[5]^ The prevalence of depression increases faster than anxiety in the early stages of the disease, increases with age and duration of the disease, and increases in prevalence throughout the course of the disease and with age^.[6]^ In patients with advanced disease, the prevalence of depressive symptoms occurs in approximately 60% of patients with advanced disease.^[7]^ Depression and PD are often interdependent, with the severity of symptoms in one affecting the course of the other. Depression in PD is associated with age, gender, freezing of gait, anxiety, fatigue, apathy, daytime sleepiness, higher doses of levodopa, higher pain scores, severity of illness, and cognitive decline.^[8]^ Patients with a PD duration of more than 10 years are at increased risk of comorbid depression.

Anxiety disorders are common but understudied in patients with PD. Anxiety complicates PD and also reduces patients’ quality of life.^[9]^ Anxiety is a common but understudied condition in patients with PD. Patients with PD are up to 40% more likely to experience anxiety symptoms, with symptoms such as generalized anxiety disorder, panic attacks, and social phobia.^[10,11]^ The symptoms include generalized anxiety disorder, panic attacks, and social phobia. The prevalence of anxiety among PD patients was found to be 68%.^[7]^ However, another study reported that as many as 45 to 49% of patients experience anxiety disorders.^[2]^ The difference between the 2 findings may be related to the assessment and diagnostic criteria, patient characteristics and comorbidities, and the stage of the disease. Anxiety in PD tends to be characterized by the following features: Anxiety manifests as an intrinsic symptom of PD.^[12]^ The prevalence of anxiety compared to depression may be more stable than that of depression.^[13]^ Anxiety is positively correlated with the severity of PD and is not significantly related to the course of the disease. Anxiety is positively associated with the severity of PD and not with the duration of the disease. Patients with PD with aggregated symptoms of postural instability and gait dysfunction are more likely to have anxiety than those with tremor predominance. Increased anxiety is associated with fluctuations in motor symptoms, particularly with dopaminergic drug depletion (i.e., the “off” period). The increase in anxiety has been associated with fluctuations in motor symptoms, particularly with dopaminergic depletion (i.e., the “off” period). This in turn increases the risk of dyskinesia or on/off fluctuations occurring. Anxiety decreases with age, with late-onset subjects having a lower prevalence than patients with young-onset PD.^[14]^ Some studies have found that risk factors for anxiety disorders in PD may be related to female gender,^[15]^ disease severity, rapid eye movement behavioral disorders, autonomic dysfunction, and dopaminergic treatment.

## 3. Pathogenesis of depression and anxiety in PD

PD depression may be related to striatal and nigrostriatal dopamine denervation, but the exact neuropathological mechanism of PD depression remains unclear. At present, PD depression cannot be explained by a single factor or mechanism but is the result of a combination of neuroanatomy, neurotransmitters, inflammation, trophic mechanisms, and psychosocial factors. Early PD depression and anxiety may also have a psychological or psychosocial component, and signs and symptoms of late disease onset may be associated with extensive neuropathology or dopaminergic treatment exposure. Genetic studies have identified genetic variants in SLC6A15, TPH2, and brain-derived neurotrophic factor, the cannabinoid 164 receptor (CB1) gene, short alleles in the 163-hydroxytryptamine transporter protein (serotonin transporter) gene linkage polymorphic region There is an association with PD depression, but several studies of serotonin and dopamine transporter protein genes have not been conclusive.^[16]^ Anatomically, depression severity was associated with lower gray matter density in bilateral orbitofrontal, bilateral rectus gyrus, right medial temporal gyrus, anterior medial and medial temporal gyrus, and parahippocampal gyrus, left hippocampus and right parahippocampal gyrus. In addition, neurotransmitter disorders play an important role, and depressive symptoms in PD are associated with reduced transporter binding in the basal ganglia (dopamine) and the blue-spot (dopamine and norepinephrine).^[17]^ Significant changes in neurotransmitter signaling (dopamine, norepinephrine, and serotonin) are also seen in limbic areas, with increased presynaptic receptor binding and decreased postsynaptic receptor binding.^[18]^ In addition, inflammatory signaling pathways,^[19]^ brain-derived neurotrophic factor,^[20]^ and polymorphisms in rs78162429 and rs1545843^[21,22]^ may be involved in the development of depressive symptoms in PD. In addition, psychological factors and motor dysfunction may be the most likely underlying mechanisms for the development of depression in PD patients.^[23]^ More in-depth studies are needed to truly understand the pathogenesis of depression in PD.

Similarly, there is still no consensus on the pathogenesis of PD anxiety disorders. It has been suggested that a short allele of the 5-hydroxytryptamine (5-HT) transporter protein gene may be responsible for the locus of inheritance of PD anxiety.^[24]^ Numerous studies have speculated that alterations in limbic cortex-striatal-thalamocortical circuits^[25]^ and dysfunction of the amygdala-insula pathway^[26]^ are risk factors for PD anxiety. There have been some studies demonstrating that deficits in the dopaminergic neurotransmitter system, 5-THergic neuronal projections at the corresponding sites,^[27,28]^ and α2-adrenergic receptors in the anterior border of the cerebral cortex are also responsible for PD anxiety. According to reports, the severity of anxiety was associated^[29]^ with alterations in fear circuits, and with the presence of interleukins (IL-1β, IL-6), tumor necrosis factor, hypersensitive C-reactive protein, soluble interleukin-2 receptor, and other receptors in the peripheral blood of patients with PD. Studies have shown that the severity of anxiety in PD patients was associated with serotonergic degeneration.^[30]^ It can be seen that PD anxiety is the result of the interaction of genetic factors, neuroanatomy, neuropathology, neurobiochemistry, and neuropsychology. More in-depth studies are still needed to truly explore the pathogenesis of PD anxiety.

## 4. Clinical manifestations of PD depression and anxiety

### 4.1. PD depression

PD depressive symptoms partially overlap with PD motor symptoms, both of which may be manifested as slow movement, hyperactivity, fatigue, unresponsiveness, and dull facial expressions.^[4]^ PD depression can occur at any time during the course of the disease. Early in the course of the disease, it may appear alone and may represent the first manifestation of PD or may contribute to the progression of PD in later stages.^[31]^ PD depression can be cognitive, autonomic, or perceptual abnormalities, can change throughout the day, and is often associated with on and off times of medication induction. It is not simple to distinguish between PD depression and primary depression, which is characterized by negative perceptions of oneself, one’s environment, and one’s future. Apathy, loss of motivation, energy and interest (both social and sexual), unresponsiveness, poor sleep and memory, reduced hunger and irritability are prominent, and self-blame and frustration are heavier than in primary depression. Some of the symptoms are more severe, such as anxiety, cognitive impairment, irritability, difficulty concentrating, and suicidal ideation without suicidal behavior, and suicidal behavior has been reported to occur in patients whose medication is withdrawn or tapered too quickly after deep brain stimulation (DBS).

### 4.2. PD anxiety

PD anxiety is characterized by fear, dread, or worry and manifests itself in the form of generalized anxiety, with panic disorder as the most common manifestation. Specific manifestations include restlessness, inability to sit still, difficulty sleeping, panic attacks, precordial discomfort, dyspnea, near-death sensations, hyperventilation, hand and foot jerks, and more. Anxiety has been found to be commonly associated with postural balance deficits, motor fluctuations, and early-onset PD^[10]^ The anxiety scores are higher in the “off” phase than in the “on” phase. Anxiety is less common in those with predominantly tremor.^[32]^ The anxiety is less common in those with predominantly tremor. Gait disturbances in patients with anxiety disorders can exacerbate anxiety and are therefore important in the treatment of anxiety disorders with respect to gait disturbances ^[10]^ disorders are therefore important in the treatment of anxiety disorders in the context of gait disorders.

## 5. Diagnosis of PD depression and anxiety

The nonmotor symptoms of PD pose distinct challenges in diagnosing psychiatric disorders. Currently, no specific diagnostic criteria exist for depression in PD. According to the 2013 Guidelines for the Diagnosis and Treatment of Depression, Anxiety, and Psychotic Disorders in PD, issued by the Neuropsychology and Behavioural Neurology Section of the Chinese Medical Association, PD patients exhibiting depressive symptoms that meet the diagnostic and statistical manual of mental disorders (DSM)-IV criteria for depression can be diagnosed with PD depression.

1. Primary PD diagnosed in accordance with the diagnostic criteria of the British PD Association Brain Bank or the Chinese PD diagnostic criteria.
2. Meets DSM-IV diagnostic criteria for depressive episodes.

It has also been reported in the literature that the 2016 diagnostic criteria for primary PD are met, but patients with PD syndrome, PD superimposed syndrome, and PD dementia should be excluded, while the diagnosis of all depressed patients meets the diagnostic criteria for depression of the Chinese classification of mental disorders-III can be diagnosed as PD depression.^[33]^ PD depression: therefore, to improve the early detection rate of PD depression, some studies advocate that once patients with PD present with depressed mood or pleasure deficiency or unexplained movement disorders, cognitive decline, or sleep disorders, they do not have to bother about whether it is attributable to PD or primary depression, and it is recommended to routinely conduct screening.^[34]^

Rating scales serve as valuable screening tools to assist clinicians in accurately diagnosing depression. A meta-analysis highlighted the 15-item Geriatric Depression Scale,^[35]^ the Beck Depression Inventory I/Ia, and the Montgomery-Asberg Depression Rating Scale as effective options for screening depression in patients with PD. However, it is important to note that the Beck Depression Inventory I/Ia is based on DSM-III criteria, and the Montgomery-Asberg Depression Rating Scale is clinician-rated rather than self-rated. Additionally, the 17-item Hamilton Depression Scale is recognized as a reliable and commonly utilized tool for assessing depression in PD patients. In particular, screening tools that include the 15-item Geriatric Depression Scale are easy to understand and contain relatively few somatically related items that overlap with core PD symptoms (sleep disturbances, altered facial expressions, and slowness), thereby reducing the impact of motor symptoms on the total score assessed.

Currently, there are no uniform diagnostic criteria for PD anxiety. Clinically, the majority of patients were found not based on clinical diagnostic criteria, but on anxiety scores. Therefore, anxiety scale assessment can not only improve the detection rate of PD anxiety. It can also assess the severity of PD anxiety. Three commonly used anxiety assessment scales are the Beck Anxiety Inventory, the Hospital Anxiety and Depression Scale, and the Hamilton Anxiety Rating Scale.^[36]^ In recent years, the Geriatric Anxiety Inventory and the PD Anxiety Scale, scales that have been developed to address PD anxiety, have stood out in the literature as reliable screening tools.^[37]^ These 2 scales have high reliability and validity but have not yet been widely used in clinical settings and require further validation. Therefore, a combination of the 2 scales can be used in the assessment and diagnosis of PD anxiety to improve the sensitivity and specificity of diagnosing PD anxiety.

## 6. Treatment of PD depression and anxiety

### 6.1. Treatment of PD depression

Despite the high prevalence of depression among PD patients, clinical research on its treatment lags far behind that of motor symptoms, and there is still a lack of clear consensus on its treatment.^[38]^ However, some current studies have found a role for nonpharmacological and pharmacological treatments in reducing depressive symptoms, although this finding is not always statistically significant.^[39–41]^

#### 6.1.1. Nonpharmacological treatment

Nonpharmacological treatments are first considered for patients with mild symptoms, and psychological interventions, physiotherapy, etc., are supplemented with treatment given to improve motor symptoms. Psychological interventions for depression, such as cognitive-behavioral therapy (CBT), are beneficial for PD. Two randomized controlled trials (RCTs) of telemedicine interventions found that 3 months of CBT (delivered via telephone or Web-based videoconferencing specific to patients with PD) was effective in improving depressive symptoms in PD compared with usual care.^[42,43]^ The use of telemedicine with video-to-home CBT has been shown to be an effective treatment when compared to conventionally based clinical treatments. A 2019 review by the Movement Disorder Society (MDS) rated CBT as potentially useful only in clinical practice, citing evidence that it may be effective but with insufficient evidence of safety. However, a recently published study of telephone-administered CBT offers a new approach to cognitive therapy for depression in PD. The advantage is that there are no significant treatment-related side effects, but it is not recommended for patients with cognitive dysfunction and speech disorders. The effects of repetitive transcranial magnetic stimulation (rTMS) on depression remain unclear.^[44]^ While some studies have reported statistically significant short-term improvements in depression scores, others have found no notable changes.^[45]^ The effect of rTMS on depression is well established. However, it was approved by the US Food and Drug Administration in 2008 for the treatment of medically refractory depression. In recent years it has been gradually applied to treat PD depression^.[46]^The 2014 European rTMS treatment guidelines state that left-sided dorsolateral prefrontal cortex high-frequency rTMS can be used as a treatment for PD depression (level B recommendation).^[47]^The 2014 European rTMS Guidelines state that left-sided dorsolateral prefrontal cortex high-frequency rTMS can be used as a treatment for PD depression (B recommendation). For patients with mild depression, CBT and rTMS, which have no obvious side effects and are well tolerated, should be considered first. For severe or refractory depression, electroconvulsive therapy (ECT) can be considered. A retrospective study found a statistically significant effect of ECT treatment, improving motor symptoms and symptoms of depression in patients with PD.^[48]^ ECT is not suitable for depressed patients with PD after DBS treatment.^[49]^

DBS is not only effective in refractory PD motor symptoms but also has a positive effect on depression in PD patients.^[50]^ Among the 2 primary DBS targets in PD, stimulation of the medial globus pallidus seems to lead to a more pronounced improvement in depressive symptoms compared to stimulation of the subthalamic nucleus (STN), even though both targets produce similar motor improvements.^[45,50]^ The degree of improvement in depression seems to be positively correlated with both the duration and severity (as measured by Unified Parkinson’s Disease Rating Scale medication scores) of PD before DBS. Consequently, patients with longer disease duration and more severe symptoms experience greater reductions in depression scores, regardless of whether they receive globus pallidus or STN stimulation. According to a recent study, depression improvements following STN-DBS were sustained for up to 2 years, with the extent of depression improvements correlating with enhancements in sleep and quality of life.^[51]^

In addition, other nonpharmacological interventions, such as bright light therapy and various forms of aerobic training, vagus nerve stimulation, are also expected to be beneficial.^[52,53]^ However, no further studies have been conducted on this therapy.

#### 6.1.2. Drug treatment

The treatment of PD depression is complex, requiring not only consideration of the relationship between depression and motor symptoms, but also attention to disease severity, as well as attention to comorbid states and other motor and nonmotor symptoms, and familiarity with therapeutic drug targets and interactions between co-administered medications. If depressive symptoms are assessed to be associated with fluctuating PD motor symptoms, it is recommended that dopaminergic medication be considered first. Clinical experience and individual small-sample clinical trials have demonstrated that levodopamine improves depressive symptoms, but its prolonged use exacerbates depressive symptoms instead.^[54]^ The effect of levodopamine on depression has been demonstrated by clinical experience and individual small-sample clinical trials, enabling the appropriate adjustment of dopamine dosage.^[55]^ When depression is not related to motor fluctuations, we need to consider the use of antidepressants to alleviate depressive symptoms. Antidepressants for Parkinson’s depression generally work through the following targets: I. 5-HT system: many antidepressants modulate the transmission and function of 5-HT by acting on 5-HT receptors, for example, 5-HT1A, 5-HT2A, etc. II. Norepinephrine (NE) system: acts on NE transporters to increase the concentration of NE in the synaptic gap. Specifically, it should be related to the regulation of neurotransmitters: increase the availability of 5-HT and NE, improve neurotransmitter imbalance, and thus alleviate depressive symptoms. Alternatively, it may involve modulating neuroplasticity by promoting neuronal growth, enhancing connectivity, and remodeling, which aids in restoring the function of damaged neural networks. Additionally, its anti-inflammatory and antioxidant properties may reduce neuroinflammation and oxidative stress, offering protective benefits to neurons.^[45]^ However, polymorphisms in genes may affect individual differences in response to antidepressant medication. Common adverse effects of antidepressant overdose in Parkinson’s depression include cardiovascular, central nervous system, gastrointestinal, metabolic, hepatic, and renal damage. A recent meta-analysis of RCTs of antidepressant medications for the treatment of major depression in PD found that current treatments primarily involve antidepressants only selective 5-HT reuptake inhibitors (SSRIs) had a statistically significant effect on depression scores. Tricyclic antidepressants (TCAs) demonstrated improved efficacy, though the results did not reach statistical significance. This contrasts with previous meta-analyses, which favored TCAs over SSRIs due to their superior efficacy. This discrepancy is significant in relation to the subgroup analyses within the meta-analysis. Additionally, when treating PD patients with SSRIs/selective 5-hydroxytryptamine and norepinephrine reuptake inhibitors (SNRIs), it is important to monitor for any worsening of extrapyramidal symptoms, although the risk remains low.^[45]^

##### 6.1.2.1. Selective 5-hydroxytryptamine reuptake inhibitors

SSRIs are commonly used as first-line treatments, and some studies have shown them to be superior to placebo.

They alleviate depression by inhibiting the reuptake of 5-HT and thereby increasing its utilization in the synaptic gap and are often recommended as first-line therapeutic agents in the treatment of depression in PD due to their low risk of overdose toxicity and ease of administration.^[56]^Citalopram and paroxetine have been reviewed by the MDS evidence-based medical evidence as potentially more advantageous in the treatment of PD depression.^[57]^ An RCT(S) confirmed that sertraline resulted in greater improvement in depression scores. Several RCTs have confirmed the effectiveness of SSRIs, but each drug also has its own unique pharmacological properties. A study conducted found that paroxetine was effective in improving patients’ depression and alleviating their cognitive function, and was safe, but side effects such as sexual dysfunction, gastrointestinal reactions, and inability to sit still may occur, possibly due to cholinergic rebound.^[58]^ Fluoxetine can be used in patients with symptoms such as excessive sleepiness, psychomotor retardation, and fatigue, and there is also the possibility of gastrointestinal and other adverse reactions. Escitalopram oxalate removes the antihistamine effect of R-citalopram and is efficacious at low doses. Overdose safety is poor, and at high doses, there is an increased risk of QT interval prolongation, as well as a possible increased risk of tremor.

##### 6.1.2.2. Tricyclic antidepressants classical antidepressants

TCA acts by blocking the reuptake of intersynaptic transmitters to enhance 5-HT, DA, and NE activity. A meta-analysis found that TCA, represented by desipramine and nortriptyline, were more effective than SSRIs in treating depression in PD,^[59]^ and were faster and more potent than SSRIs. The other meta-analysis also found that TCAs and SSRIs were superior to SSRIs in the treatment of depression in PD,^[41]^ and although TCAs may improve depression in PD, adverse effects such as cardiotoxicity, sedation, constipation, blurred vision, and postural hypotension need to be noted, and cognitive impairment may be exacerbated due to its anticholinergic effects, while the potential for lethality of an overdose in the elderly needs to be taken into account. The adverse effects of TCA have limited its clinical use, and it is mostly used when SSRIs are ineffective.

##### 6.1.2.3. Selective 5-hydroxytryptamine and norepinephrine reuptake inhibitors

SNRIs have dual reuptake inhibitory effects of 5-HT and NE. They can not only treat depressive symptoms but also relieve anxiety and improve somatic pain symptoms, and with their good safety and tolerability, they can be used as the first-line treatment for depression. They are represented by duloxetine and venlafaxine. A study conducted found that venlafaxine was effective in relieving PD depression without increasing motor symptoms, but it should be avoided at night or in patients with insomnia, and overdose of venlafaxine has been shown to cause adverse effects such as increased blood pressure and increased heart rate. A 12-week RCT(S) showed a unique effect of duloxetine in depressed patients with pain.^[60]^ A small RCT(S) of duloxetine also found that it was able to reduce the “off phase” in some patients,^[61]^ and that the adverse effect of overdose may also be an increase in blood pressure.

##### 6.1.2.4. Dopamine agonists

One of the pathways involved in the dopamine system is the mesolimbic-limbic pathway, which is associated with reward, pleasure, and motivated behavior. The physiological function of this pathway is most relevant to depression, and its dysregulation may result in a lack of pleasure, impaired motivation, and psychomotor developmental delays. PD patients have degenerative deficits of dopaminergic neurons in the brain, resulting in decreased dopamine levels. Dopamine agonists can directly stimulate dopamine receptors and mimic the effects of dopamine, thereby supplementing the dopaminergic neurotransmitters and improving motor symptoms and depression. Pramipexole, although primarily used to treat motor symptoms in PD, has also been studied for use as a mood elevator in PD. Pramipexole was found to better improve depression scores in an open RCT(S) study.^[62]^ Dopamine agonists such as ropinirole and rotigotine have been less frequently studied, primarily as adjunct therapies and their phasic efficacy remains uncertain. The therapeutic role of dopamine agonists in PD depression is still under investigation. However, excessive dopamine agonists are prone to psychotic symptoms, hallucinations, impulse control disorders, etc., which still need our close attention.

##### 6.1.2.5. Monoamine oxidase B inhibitors. Monoamine oxidase B

(MAO-B) inhibitors lie in the alleviation of motor symptoms by blocking the degradation of dopamine in the brain, thereby increasing dopamine concentrations. As a representative drug, safinamide has a higher selectivity for MAO-B and is reversible with a wider safety profile. A 2-year study showed statistically significant improvements in PDQ-39 emotional health scores and Global Rating of Illness Deficits-Hamilton Depression Scale scores at 6 and 24 months in the safinamide group.^[63]^ However, due to the increased risk of autonomic dysfunction and hypertensive crises associated with monoamine oxidase inhibitors, caution is recommended when using these treatments.^[45]^

##### 6.1.2.6. Other new possible antidepressants

Another adjunctive therapy explored was omega-3 fatty acid supplementation. A report from a double-blind RCT involving 29 PD patients with depression found a statistically significant improvement in depression scores among those using omega-3 fatty acids.^[64]^ ω-3 fatty acids in the treatment of Parkinson’s depression and anxiety may be a variety of mechanisms of action together, such as anti-inflammatory effects, neuroprotection, regulation of neurotransmitters, improve cell membrane function, regulation of gene expression, antioxidant effects, etc., but the specific mechanism of action needs further in-depth study to come to elucidate. The current murine model of PD depression has identified several potential therapies for depression, but these items are still under further study. A growing number of studies have found that continuous infusion therapy with levodopa/carbidopa enteric gel or subcutaneous apomorphine may improve depressive symptoms in addition to important benefits for motor symptoms and quality of life.^[65]^ Istradefylline, a novel adjuvant approved by the FDA for the treatment of motor symptoms in PD disease, has been shown to improve PD depression scores.^[66]^ The mechanism may occur in a 5-HT-independent manner via adenosine A2A receptor antagonism, suggesting that it may have a faster onset of action than 5-hydroxytryptaminergic medications and is a safe adjuvant.^[67]^ Ketamine, an *N*-methyl-D-aspartate receptor antagonist, is sometimes utilized for refractory depression in the general population. In mouse models of PD and case reports, it has been shown to alleviate depressive symptoms through the modulation of glutamate and brain-derived neurotrophic factor-mediated increases in synaptic plasticity, though monoamines might also contribute to the effects.^[68]^ The antidiabetic drug pioglitazone has been studied and found to have an ameliorative effect on a murine model of PD depression. It is believed to act through its anti-inflammatory properties in the hippocampus, reducing microglial activation and promoting neurogenesis in hippocampal neurons, which are thought to contribute to depression in PD.^[69]^ Likewise, melatonin is thought to alleviate PD-related depression through its neuroprotective, antioxidant, and neurotrophic effects.^[45]^ These properties have been shown to improve both motor and depressive symptoms, as well as sleep disturbances associated with circadian rhythm disruptions in murine PD models.^[70]^ In conclusion, it is hoped that there will be more research and experiments on PD depression to help address depression in PD patients.

Regardless of the type of pharmacological treatment, once a drug has been selected for monotherapy, the dose should be adjusted to the lowest effective dose within 2 to 3 weeks, depending on the patient’s tolerance, and then the patient should be allowed to respond within 4 to 8 weeks. If the patient does not respond, the dose is increased as tolerated by the patient until symptoms improve or the maximum dose is given. The general principles of drug administration are to start small, ramp up slowly, monitor closely for associated side effects, and perform periodic efficacy assessments.^[71]^ When severe depression or suicidal tendency is clearly identified, immediate psychiatric counseling is recommended, or multidisciplinary joint treatment with neurology, psychiatry, and rehabilitation nursing team is given. In addition, care and attention from the family also play an important role.^[72]^ The family also plays an important role.

### 6.2. Anti-anxiety treatment

Although anxiety is a prevalent factor contributing to the poor quality of life in PD patients, to date there are no placebo-controlled randomized clinical trials using anxiety symptoms as a primary outcome indicator and evidence-based medical evidence for anxiety treatment in PD is lacking. There are also no RCT(s) evaluating any pharmacological or nonpharmacological treatments. However, in some RCT(S) of PD depression treatment studies, depression and anxiety usually occur concomitantly and anxiety indicators are considered secondary outcomes. Some studies have concluded that treatment from a psychological perspective can improve PD anxiety patients’ perceptions of health, illness, etc., and thus further improve their anxiety symptoms.^[73]^ In a placebo-controlled and nonplacebo-controlled RCT(S) comparing the use of CBT and no intervention for depression in PD, secondary measures of anxiety showed a significant sustained improvement in anxiety scores within 6 months of treatment.^[74]^Another nonpharmacological intervention that may be considered for patients with PD early in the course of the disease is exercise therapy, as aerobic exercise has been shown to have anxiolytic effects in the general population.^[75]^ However, this approach has yet to be studied in PD patients. In addition, when used for motor symptoms, DBS seems to provide a small short-term reduction in anxiety, although anxiety levels tend to return to baseline over time.^[76,77]^

Recent studies have found that transcranial magnetic stimulation studies have some therapeutic effects on PD anxiety. A study performed low-frequency stimulation of the ventral medial prefrontal cortex in patients with PD anxiety for 14 days of continuous treatment, and the patient’s anxiety symptoms improved significantly^.[78]^ The mechanism may be through the stimulation of brain nerves, which can change the neural response and transmitter release. However, this study has some limitations, lack of long-term follow-up observation, as well as long-term safety and efficacy, which need to be confirmed by further research. ECT has been a successful treatment for depression and anxiety symptoms, with high safety and efficacy, and no adverse events have been reported.

There is a lack of evidence-based medical evidence for the pharmacological treatment of patients with anxiety in PD, which is closely related to dyskinesia and often occurs during the “off phase,” when pharmacological treatments targeting the “off phase” can help to alleviate anxiety symptoms.^[79]^ The evidence suggests that PD anxiety is closely related to dyskinesia. Considering that PD anxiety is associated with depression, antidepressant treatment can improve anxiety symptoms. It was found that SSRIs (citalopram), TCAs (nortriptyline and desipramine), and atomoxetine had statistically significant effects on secondary anxiety indicators, while paroxetine showed a moderate effect that was not statistically significant.^[80]^The 5-HT reuptake inhibitor class of drugs needs to be taken seriously because of its predilection for adverse effects and risky use. It has the potential to cause adverse effects such as nausea, vomiting, drowsiness, and in severe cases, worsening tremor. Long-term administration carries risks of hyponatraemia, sexual dysfunction, and weight changes.

In cases of severe anxiety, benzodiazepines have some value, but it should be noted that they are best avoided in the clinic due to the increased risk of causing autonomic dysfunction, and fractures from falls, sedation, and exacerbating cognitive dysfunction.

## 7. Limitations of the study

In the process of reviewing the latest advances in PD depression and anxiety, we found that there are some limitations that cannot be ignored. Firstly, in terms of research methodology, there is a bias in the selection of samples, with some studies focusing on patients in specific healthcare organizations or regions, which may result in findings that do not accurately reflect the real situation of patients with depression and anxiety in PD globally. In addition, inconsistencies in diagnostic criteria make comparisons and integration between different studies challenging, affecting our understanding of the consistency of disease diagnosis.

In terms of research content, the exploration of pathogenesis still needs to be deepened. Although multiple hypotheses have been proposed, how various factors interact to cause depression and anxiety in PD has not yet been fully clarified. In treatment studies, there is a relative lack of data on the long-term effects and safety of pharmacological treatments, and large-scale, long-term follow-up studies of nonpharmacological treatments are rare. At the same time, there is a lack of studies that take into account various aspects of patients’ quality of life and social support, limiting our comprehensive understanding of the overall management of the disease.

In summary, despite significant progress in the study of depression and anxiety in PD, there is still a need to overcome these limitations to advance the field further and provide more effective diagnostic and therapeutic approaches for patients.

## 8. Conclusion

Depression and anxiety represent prevalent comorbidities associated with PD. Over the past few years, research into these psychiatric conditions has achieved considerable advancements. The current pathogenesis associated with PD depression and anxiety should be the result of a combination of neuroanatomy, neurotransmitters, inflammation, trophic factor mechanisms, and psychosocial factors and still needs further research. Although there is a lack of uniform guidelines, we still need to be alert to the emergence of PD depression and anxiety based on the Anxiety Depression Scale, early intervention, and individualized pharmacological or nonpharmacological treatment. Future research, with multidisciplinary collaboration and more large-scale, randomized, double-blind, placebo-controlled trials in-depth, could provide more valuable evidence on medication use and introduce new effective pharmacological and nonpharmacological treatment modalities, develop more accurate diagnostic methods and more effective treatment strategies, and improve patients’ quality of life.

## Author contributions

**Writing—original draft:** Zhonglong Wang.

**Writing—review & editing:** Zhonglong Wang, Wei Qin.

**Methodology:** Hongkai Wei.

**Validation:** Yanhong Xin.
